# Sustained delivery of 17β-estradiol by human amniotic extracellular matrix (HAECM) scaffold integrated with PLGA microspheres for endometrium regeneration

**DOI:** 10.1080/10717544.2020.1801891

**Published:** 2020-08-05

**Authors:** Yue Chen, Weidong Fei, Yunchun Zhao, Fengmei Wang, Xiaoling Zheng, Xiaofei Luan, Caihong Zheng

**Affiliations:** Department of Pharmacy, Women’s Hospital, School of Medicine, Zhejiang University, Hangzhou, China

**Keywords:** Human amniotic extracellular matrix, biologic scaffolds, PLGA microspheres, endometrium regeneration

## Abstract

The endometrial injury usually results in intrauterine adhesions (IUAs). However, there is no effective treatment to promote the regeneration of the endometrium currently. The decellularized amnion membrane (AM) is a promising material in human tissue repair and regeneration due to its biocompatibility, biodegradability, as well as the preservation of abundant bioactive components. Here, an innovative drug-delivering system based on human amniotic extracellular matrix (HAECM) scaffolds were developed to facilitate endometrium regeneration. The 17β-estradiol (E_2_) loaded PLGA microspheres (E_2_-MS) were well dispersed in the scaffolds without altering their high porosity. E_2_ released from E_2_-MS-HAECM scaffolds *in vitro* showed a decreased initial burst release followed with a sustained release for 21 days, which coincided with the female menstrual cycle. Results of cell proliferation suggested E_2_-MS-HAECM scaffolds had good biocompatibility and provided more biologic guidance of endometrial cell proliferation except for mechanical supports. Additionally, the mRNA expression of growth factors in endometrial cells indicated that HAECM scaffolds could upregulate the expression of EGF and IGF-1 to achieve endometrium regeneration. Therefore, these advantages provide the drug-loaded bioactive scaffolds with new choices for the treatments of IUAs.

## 1. Introduction

Intrauterine adhesions (IUAs), known as Asherman syndrome, are characterized by damage to the endometrium due to curettage or endometritis (Dreisler & Kjer, 2019). IUAs cause endometrial functional repair disorder leading to endometrial fibrosis. Approximately 25–30% of infertile women suffer from IUAs, which represents the most common cause of uterine infertility (Hanstede et al., 2015). It is also associated with the following gynecological diseases: hypomenorrhea, amenorrhea, recurrent abdominal pain, and recurrent spontaneous abortion. The traditional treatments for IUAs mainly focus on hysteroscopic transcervical resection of adhesion (TCRA) combined with postoperative management, including prevention of adhesion reformation (by the placement of an intrauterine device, foley catheter balloon, biomaterials, or other methods), and estrogen therapy for stimulation of endometrial regeneration (Khan & Goldberg, 2018). However, the recurrence of IUAs is still prevalent after various treatments, in severe cases, the incidence of adhesion reformation was reported to be as high as 62.5% (Rein et al., 2011). In addition, there are some disadvantages in available IUA treatments, including high recurrence rate, low pregnancy rate, and increased risk of thrombosis and breast tumors due to the high therapeutic dose of estrogen (Brown & Hankinson, 2015). Thus, it is highly desired to develop alternative approaches that lead to endometrial functional repair.

Tissue regeneration is considered to be a promising way for the treatment of IUAs. Recently, natural tissue-derived biomaterials have been increasingly applied to prevent postoperative re-adhesion (Kou et al., 2020). The amnion membrane (AM), a kind of prospective biomaterials, could not only act as a mechanical barrier to separate the wounded surface but also secrete various growth factors to activate the regulation of endometrial cell proliferation, migration, differentiation to achieve the morphological and functional recovery of the uterus (Shakouri-Motlagh et al., 2017). In the clinic, both the fresh and lyophilized amnion membranes have been used as anti-IUAs therapy (Cai et al., 2017; Li et al., 2020). However, AM may induce an immune response when integral membranes are transplanted into the majority of human tissues. Decellularized AM can reduce potential immunogenicity as the extracellular components of tissues are well tolerated even when used as xenografts (Aamodt & Grainger, 2016). In addition, by eliminating cellular components, the extracellular matrix (ECM) is more exposed, which promotes tighter cell-ECM interactions and results in more efficient cell attachment as well as stimulating different cell behaviors (Portmann-Lanz et al., 2007; Shakouri-Motlagh et al., 2017). ECM-derived scaffolds have been applied to promote the regeneration of various tissues in both preclinical and clinical types of research (Nogami et al., 2016; McQuilling et al., 2019), but there are no reports about the treatments of IUA with human amnion extracellular matrix (HAECM).

**Figure 1.**
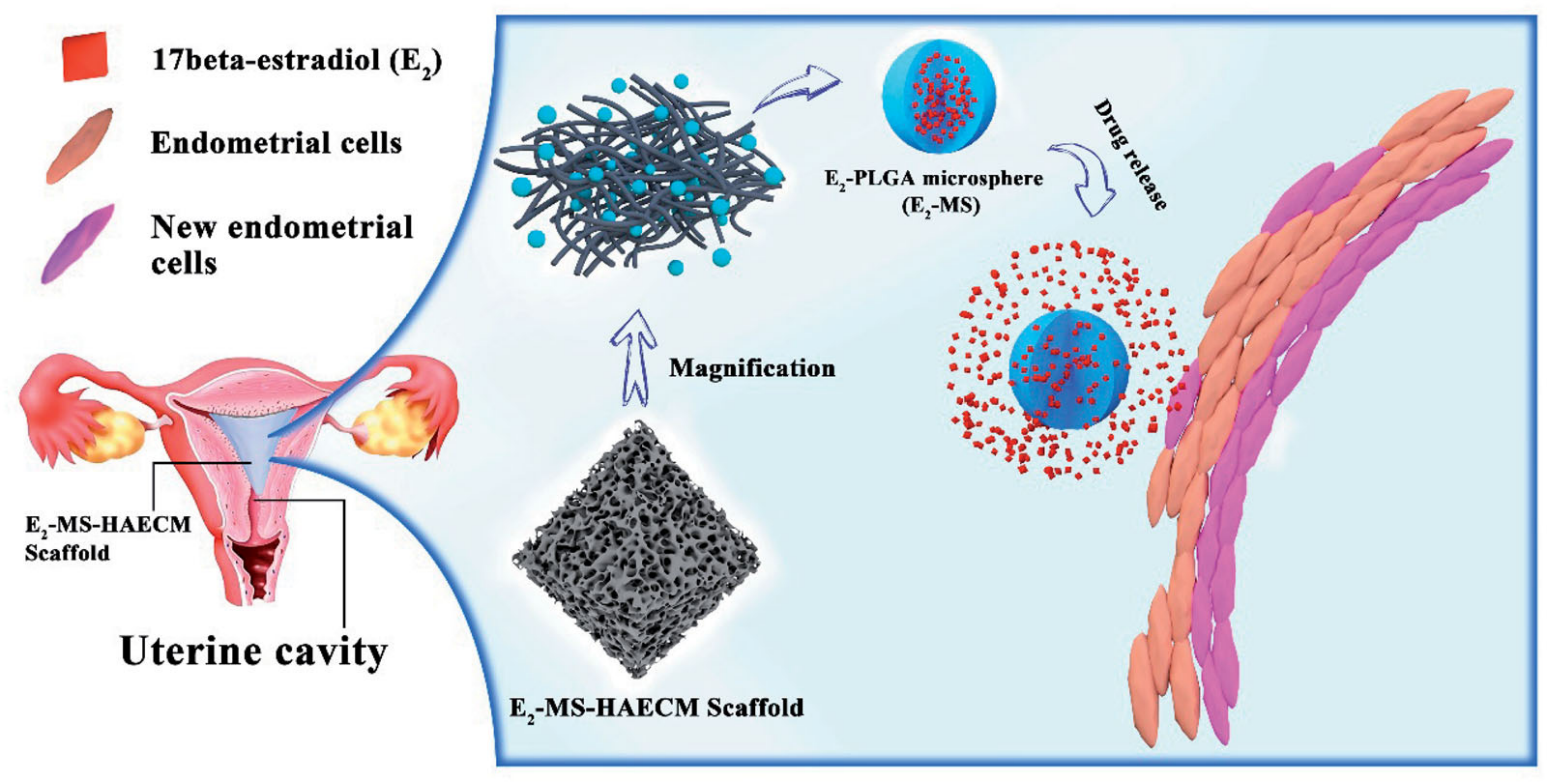
Schematic diagram of E_2_-MS-HAECM scaffold as the intrauterine controlled release system for endometrium regeneration.

In order to improve the intrauterine drug concentrations of estrogen and reduce the severe side effects caused by high oral dose, we designed a kind of intrauterine drug sustained-release system based on HAECM bioscaffolds. Poly lactic-co-glycolic acid (PLGA) is an attractive polymer material due to its high biocompatibility, biodegradability, and excellent safety. PLGA microspheres have been demonstrated as effective carriers for estrogen in the drug delivery system (Sun et al., 2008). Zhang et al. reported that the application of 17β-estradiol (E_2_) released from the uterus improves the therapeutic effect for IUA in a rat model (Zhang et al., 2017). Hence, as shown in Figure 1, intrauterine HAECM-derived scaffolds embedded E_2_-PLGA microsphere (E_2_-MS) is introduced to be an efficient way to promote endometrial regeneration.

This work presents the first attempt to prepare a drug-delivering system based on HAECM scaffolds. Physicochemical properties of the scaffolds such as main composition, gelation kinetics, rheology, morphology, and drug release *in vitro* were investigated. Their biological properties, such as cytotoxicity, cell proliferation on the scaffolds, were evaluated to examine whether the scaffold composite could support endometrial cell adhesion, and growth. At last, the gene expressions of EGF, IGF-1, and their respective receptors in endometrial cells were analyzed after exposure to treatment groups.

## 2. Materials and methods

### 2.1. Materials

PLGA Resomer^®^ RG 502 H (L:G 50:50, viscosity 0.16–0.24 dl/l, 7000–17,000 Mw), PLGA Resomer^®^ RG 503 H (L:G 50:50, viscosity 0.32–0.44 dl/l, 24,000–38,000 Mw), and 17β-estradiol (E_2_) were was purchased from Sigma-Aldrich. 3-(4,5-dimethyl-2-thiazolyl)-2,5diphenyl-2-H-tetrazolium bromide (MTT) and tritonX-100 lysis buffer were obtained from Sigma-Aldrich (St. Louis, US). Minimum Eagle’s medium (MEM), fetal bovine serum (FBS), glutamine, penicillin G sodium, and streptomycin sulfate were obtained from Gibco (BRL, Gaithersburg, US). All other solvents and reagents were of analytical grade and used without further purification.

### 2.2. Cell culture

Ishikawa cells were provided by the Women’s Hospital School of Medicine Zhejiang University (Hangzhou, China). Ishikawa cells were cultivated in MEM supplemented with 5% FBS (v/v), 2 mM glutamine, 100 IU/ml penicillin G sodium and 100 mg/ml streptomycin sulfate at 37 °C with 5% CO_2_ in a humidified incubator.

### 2.3. Amniotic membrane (AM) and decellularization

Term placentas with attached fetal membranes were collected immediately after uncomplicated cesarean section from healthy women, being tested negative for HIV, Hepatitis B and syphilis. The study and the use of the AM were approved by the Ethics Committee of the Women’s Hospital School of Medicine Zhejiang University.

AM samples were carefully pulled apart from the chorion under sterile conditions, and the chorion was discarded. The membrane was washed several times with PBS containing 50 mg/ml penicillin and 50 mg/ml streptomycin to remove blood and blood clots. The membrane was cut into several 10 cm*10 cm pieces and agitated in 0.1% TritonX-100 for 6 h in room temperature, followed by rinsing with sterile PBS for 15 min. Next, the tissue was incubated in 0.25% Trypsin–EDTA solution overnight for cell removal. In order to eliminate nucleic acids completely, the tissues were then incubated in 1.8 M sodium chloride (NaCl) for 12 h to disrupt cell membranes and nuclear components. Finally, it was washed thoroughly with PBS, lyophilized and stored at 4 °C until use.

### 2.4. Histology of HAECM

To examine the extent of decellularization of the amniotic membrane, both fresh and decellularized samples had been embedded in O.C.T compound after fixing in 4% paraformaldehyde, then sectioned at 10 μm thickness using a microtome. Hematoxylin and eosin (H&E) stains were applied for histological analysis.

### 2.5. Hydrogels formation and glycosaminoglycans (GAGs), collagen analysis

The lyophilized decellularized AM was ground into powder at low temperatures and digested by 1 mg/ml pepsin in 0.1 M HCl solution followed by agitation on a shaker at 250 RPM for 72 h at room temperature. The digestion was stopped by pH adjustment to 7.2-7.4 using 0.1 M NaOH. The polymerization of neutralized HAECM hydrogel was initiated by incubation at 37 °C for a certain time.

The concentration of sulfated glycosaminoglycans (GAGs) in native AM and the neutralized HAECM hydrogel (5 mg/ml) were measured by the sulfated GAG assay kit (Biocolor, UK). The concentration of acid-pepsin soluble collagens (Types I to V) was determined by soluble collagen assay (Biocolor, UK). Three samples were picked for each assay.

### 2.6. HAECM hydrogel rheology and turbidimetric gelation kinetics

Gelation kinetics were determined by measuring the change in turbidity of various fibrin solutions. For each group, 150 μl/well of HAECM gel was added in a 96-well plate. The turbidity of hydrogels was measured by spectrophotometry with a microplate reader (Thermo MK3, US) at 550 nm every two mins for 60 mins at 37 °C.

The rheological characteristics of HAECM hydrogels were determined with a rotational rheometer (RS6000, HAAKE, Germany) operating with a 50 mm parallel plate geometry. The temperature was set to 37 °C, and the HAECM hydrogels with different concentrations were prepared and loaded into the rheometer. To obtain the dynamic viscosity, storage and loss modulus of the hydrogels, a dynamic time sweep test with a frequency of 1 rad/s and a strain of 1% was applied for 40 min.

### 2.7. Preparation of 17β-estradiol-loaded PLGA microspheres (E_2_-MS)

E_2_-MS were prepared by O/W emulsion-solvent extraction/evaporation method. We applied two PLGA materials: PLGA Resomer^®^ RG 502 H (L:G 50:50, viscosity 0.16–0.24 dl/l, 7000–17,000 Mw), PLGA Resomer^®^ RG 503 H (L:G 50:50, viscosity 0.32–0.44 dl/l, 24,000–38,000 Mw). Due to the lower solubility of estradiol in dichloromethane, ethyl acetate was selected as the organic phase. Briefly, 400 mg of PLGA and 40 mg of E_2_ were dissolved in 4 ml ethyl acetate. The organic phase was added dropwise to 40 ml of the inner aqueous phase containing 1.5% PVA. The entire mixture was emulsified by homogenization at 10,000 rpm for 2 min. The resulting emulsion was then added to 600 ml of distilled water and stirred at 250 rpm for 24 h at room temperature. The collected microspheres were washed three times in water and then lyophilized for 48 h (Labconco, FreeZone, Kansas City, MO) and stored −20 °C before further analysis.

### 2.8. Drug loading efficiency and entrapment efficiency

The concentrations of E_2_ in PLGA microspheres were measured by the HPLC method on Agilent 1100 series system (Agilent, Santa Clara, CA), consisted of a pump and a UV-Vis detector set at 280 nm. The analytical column was a C_18_ column (4.6 mm × 250 mm, 5 μm) (Agilent). The HPLC mobile phases consisted of methanol and water (65:35, v/v) at a flow rate of 1.0 ml/min. The injection volume was 20 μl. The quantification of E_2_ was performed using a resulting standard curve with the transformation of peak areas into the concentrations.

About 10 mg of the freeze-dried microspheres were dissolved in 1 ml of dichloromethane. The obtained solution was then diluted to 10 ml using methanol and vortex-mixed for 5 min. 1 ml of the dispersion was centrifuged at 8000 rpm (TGL-16G centrifuge, China) for 10 min. The concentrations of E_2_ were determined by taking the supernatant for HPLC system analysis. The drug loading efficiency (LE) and encapsulation efficiency (EE) were calculated by the following formulae:

(1) LE%=(Drug amount in microspheres      /Total amount of PLGA microspheres)× 100

(2) EE%= (Drug amount in microspheres/Total drug amount)×100

### 2.9. Preparation of porous HAECM scaffolds and HAECM scaffolds integrated with E_2_-MS

After gelation at 37 °C, the HAECM hydrogels with two different concentrations (5 mg/ml and 8 mg/ml) were frozen slowly in an isopropanol bath at −20 °C for 12 h and lyophilized. Next, the scaffolds were rehydrated in PBS at 37 °C for 12 h and rinsed with deionized water. The scaffolds were then lyophilized again and stored −20 °C.

E_2_-MS was dispersed in the HAECM hydrogels with two different concentrations (5 mg/ml and 8 mg/ml) to form the final microsphere-HAECM composite hydrogels at 37 °C. The final weight ratio of the microspheres was controlled as 30% of the total complex mass. Finally, porous complex scaffolds were prepared following the lyophilization protocol mentioned above.

The mean porosity of the different scaffolds was calculated as the relation between the void volume and the initial volume before lyophilizing.

### 2.10. Morphologic analysis

The shape and surface morphology of E_2_-MS, HAECM scaffolds, and microspheres-HAECM scaffolds were observed by a scanning electron microscope (SU8220 FEI Quanta 650, Livonia, MI). The samples of scaffolds were coated by ion sputter gold under vacuum. The surface and structure morphology were analyzed, and the pictures were taken.

### 2.11. Drug release in vitro

#### 2.11.1. Drug release from E_2_-MS

*In vitro* drug release from E_2_-MS was performed using the dialysis membrane method. The dialysis bags (cutoff Mw 8000–14,000, Sigma) were soaked in deionized water for 12 h before use. About 20 mg E_2_-MS were placed in a dialysis bag containing 1.5 ml of PBS (pH 7.4) and immersed into a flask with 40 ml of PBS (pH 7.4), which was shaken at 60 rpm. At the settled time point, 2 ml dissolution medium was withdrawn and an equal volume of fresh medium was added to the flask to maintain a constant volume. Then the samples were centrifuged at 10,000 rpm for 10 min. The amounts of E_2_ in the supernatant were analyzed by the HPLC system.

#### 2.11.2. Drug release from E_2_-MS-HAECM scaffolds

*In vitro* drug release from lyophilized scaffolds was measured by ultrafiltration. 200 mg scaffolds (with two different concentrations: 5 mg/ml and 8 mg/ml) loaded with 30% E_2_-MS was placed into an ultrafiltration centrifuge tube (cutoff Mw 10,000, Millipore), and 10 ml PBS (pH 7.4) was added. The tube was shaken with 60 rpm at room temperature. At specific time points, the entire medium was removed and replaced by 10 ml fresh PBS. The samples were centrifuged at 10,000 rpm for 10 min. The amounts of E_2_ in the supernatant were analyzed by the HPLC system.

### 2.12. Ishikawa cells growth with E_2_-MS, free HAECM scaffolds, and E_2_-MS HAECM scaffolds

In order to evaluate the response of Ishikawa cells to different E_2_ concentrations, 10^4^ cells were seeded on 96-well plates for 24 h and treated with E_2_ with different concentrations (0, 10, 100, 1000, and 10,000 nM) in triplicates. The proliferation of cells was evaluated by MTT reduction method after incubation for 24, 48, and 72 h. In brief, the cells were stained with MTT for 4 h and then dissolved in DMSO. Optical density (OD) was read with a microplate reader (Thermo MK3, Waltham, MA) at 570 nm. Cell proliferation was expressed as the ratio between the amount of formazan determined for cells treated with E_2_ of different concentrations and for nontreated cells.

For determining the effect of E_2_-MS, free HAECM scaffolds, and microspheres-HAECM complex scaffolds on Ishikawa cells, appropriate doses of E_2_-MS or scaffolds were added to each well on 6-well plates. After incubation for 24, 48, and 72 h, the cells were stained with MTT for 4 h and then dissolved in DMSO. A 100 μl solution was extracted from each well and transferred to a 96-well plate. The cell viability was assessed using the MTT assay as mentioned above.

### 2.13. RNA isolation and quantitative real-time reverse transcription PCR (qRT-PCR) analysis

After incubation with different preparations for 72 h in 6-well plates, total mRNA from Ishikawa cells was extracted using the Trizol reagent. RNA was reversed to cDNA using a PrimeScript Reverse Transcription (RT) reagent kit (Takara) following the instructions. Specific primers used for amplification were synthesized by Generay (Shanghai, China; Table 1). Based on the SYBR Premix Ex Taq^TM^ kit (Takara, Japan), qRT-PCR reaction was performed with an ABI 7500 Thermocycler (Applied Biosystems, USA). For each sample, an average cycle threshold (Ct) value was calculated from triplicate wells, and GAPDH was used as the control of the input RNA level. The relative gene expression was calculated using 2^−△△Ct^ method.

**Table 1.** Primer sequences for quantitative real-time PCR.

Target gene	Primer Sequence
*GAPDH*	
Forward	5′-CTC ATG ACC ACA GTC CAT GC-3′
Reverse	5′-TTC AGC TCT GGG ATG ACC TT-3′
*EGF*	
Forward	5′-TGT TTC CTG TCC ACG CAA TG-3′
Reverse	5′-TGG GCT AAG AGG AAC GCA GA-3′
*EGFR*	
Forward	5′-CGA CCA AAG TTC CGT GAG TT-3′
Reverse	5′-ATC CAC CAC GTC GTC CAT GT-3′
*IGF-1*	
Forward	5′-TCA GCA GTC TTC CAA CCC AA-3′
Reverse	5′-AAG GCG AGC AAG CAC AGG-3′
*IGF-1R*	
Forward	5′-CCA GCG TTA TGA GAT CAA GA-3′
Reverse	5′-AGT ATC CGC AGA CAC TCT CC-3′

### 2.14. Statistical analysis

Data are expressed as mean ± SD, and the statistical comparisons of means were performed by standard analysis of Graphpad prism 7.0. A *p*-value <.05 was considered statistically significant in all cases.

## 3. Result

### 3.1. Histology of amniotic membrane (AM) and decellularized AM

**Figure 2.**
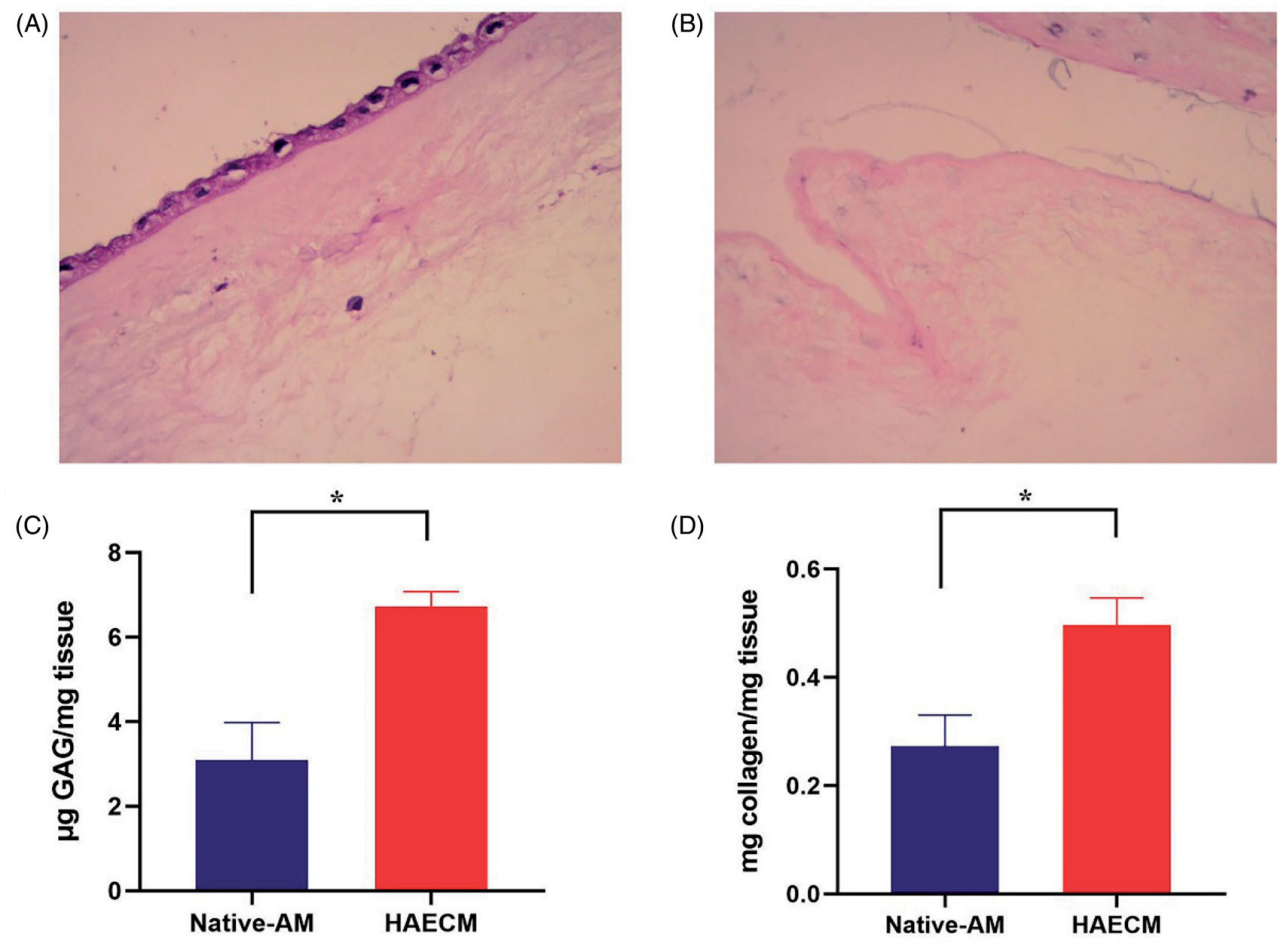
H&E stain of native AM (A) and decellularized AM (B). (C) Quantification of sulfated GAGs in the native AM and the HAECM hydrogel. Results are presented as mean μg sulfated GAGs per mg matrix. (D) Quantification of collagen in the native AM and the HAECM hydrogel. Results are presented as mean mg collagen per mg matrix. Data are presented as mean ± standard deviation; *n* = 3.

HE stains reveal that the AM is consists of three major layers: a single layer of columnar epithelial cells, attached to an underlying dense basement membrane, an acellular compact collagen-rich layer underneath, and finally a layer of dispersed fibroblasts (Figure 2(A)). The lack of staining for these cellular components further supported our successful decellularization results (Figure 2(B)).

### 3.2. Glycosaminoglycans (GAGs) and collagen analysis

Our research measured the content of GAGs between native AM and HAECM hydrogel. The native AM contained 3.09 ± 0.885 μg sulfated GAGs per mg tissue, while HAECM containing 6.72 ± 0.356 μg GAGs per mg hydrogel (Figure 2(C)). The concentration of GAGs in HAECM hydrogel was significantly higher than that of native AM (*p* < .05). The soluble collagen content in HAECM hydrogel with a concentration of 5 mg/ml was 0.497 ± 0.05 mg per mg hydrogel, while the native AM contained 0.273 ± 0.057 mg soluble collagen per mg tissue (Figure 2(D)), and there were significant differences among the different concentration of hydrogel (*p* < .05).

### 3.3. Gelation kinetics and rheological properties

**Figure 3.**
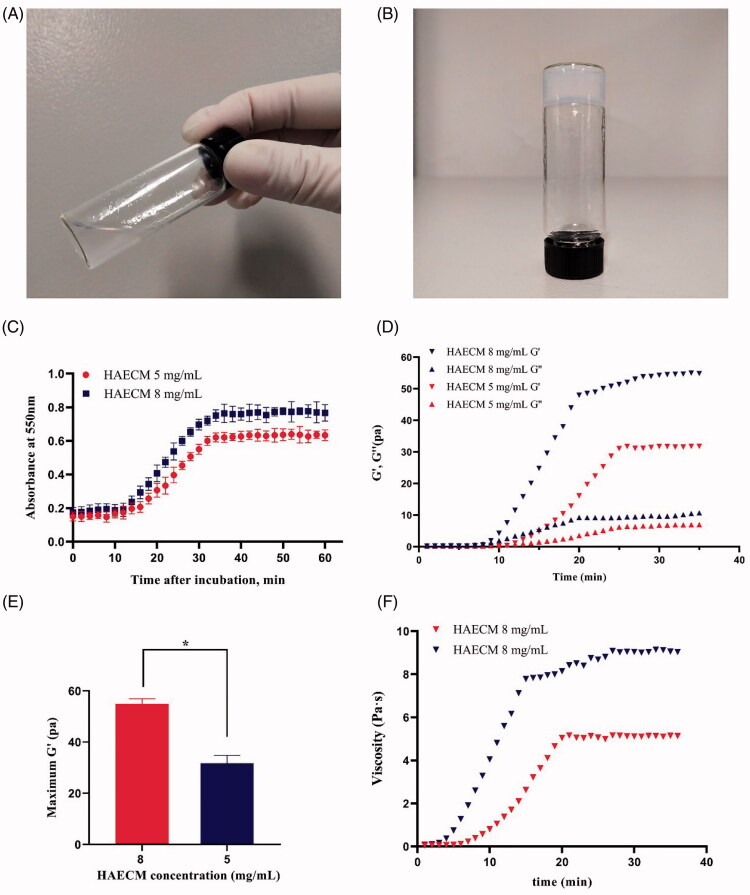
The state of HAECM hydrogel at different temperatures (15 °C (A) and 37 °C(B)). (C) Turbidimetric gelation kinetics of hydrogels. Hydrogel absorbance was measured spectrophotometrically at 550 nm. (D) Representative curves of the gelation kinetics of HAECM hydrogels based on storage and loss modulus (G′ and G′′). (E) The maximum storage modulus (G′) after complete gelation for each hydrogel as a function of ECM concentration. (F) The dynamic viscosity of hydrogel was determined under constant stress after inducing gelation. Data are presented as mean ± standard deviation; *n* = 3.

HAECM hydrogels achieved 95% of its rigidity within 32 min after incubation initiation at 37 °C (Figure 3(C)). The increase in the concentration of hydrogels (8 mg/ml versus 5 mg/ml) played a role in physical gel density but had no effect on gelation time.

As shown in Figure 3(D), both the storage modulus (G′) and the loss modulus (G′′) changed over time and were characterized by a sigmoidal shape when the temperature of the samples was set to 37 °C. G′ and G′′ reached a steady-state after approximately 30–35 min, suggesting that gelation has been completed. Solid like behavior was confirmed because the G′ was about 5 times greater than the G′′ after gelation. HAECM hydrogels showed an increase in the rate of gelation with increasing concentration. Indeed, the maximum G′ of the 8 mg/ml hydrogel was significantly higher than that of the 5 mg/ml hydrogel (*p* < .05) after gelation was completed (Figure 3(E)). As shown in Figure 3(F), the dynamic viscosity of each hydrogel was determined after inducing gelation, of which the change tendency was consistent with the result of G′ and G′′ study.

### 3.4. Characterization of HAECM scaffolds and scaffolds integrated with E_2_-MS

**Figure 4.**
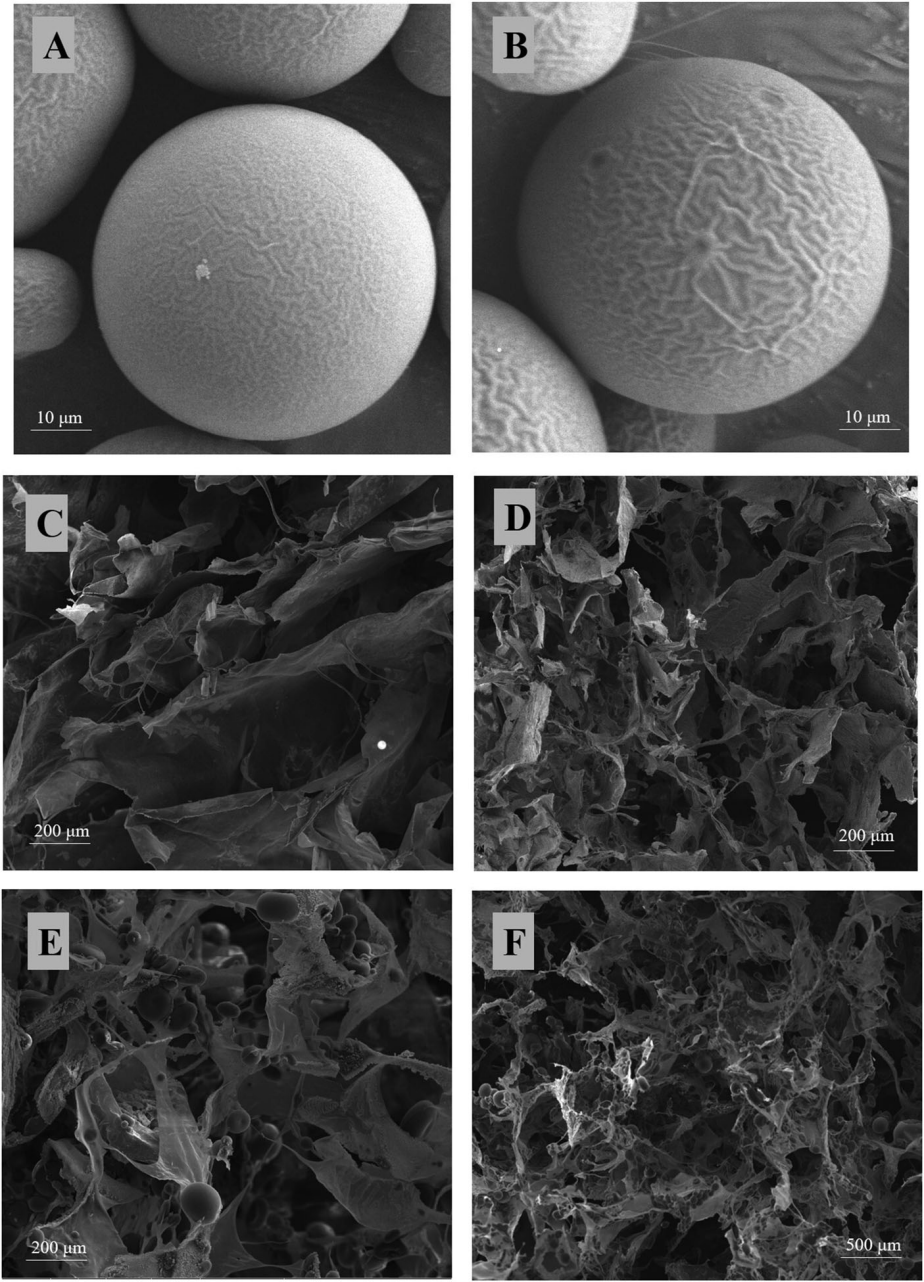
Scanning electron microscopy photograph. (A) E_2_-MS (502H). (B) E_2_-MS (503H). (C) HAECM scaffold (5 mg/ml). (D) HAECM scaffold (8 mg/ml). (E) E_2_-MS-HAECM scaffold with high magnification. (F) E_2_-MS-HAECM scaffold with low magnification.

The decellularized AM was digested into liquid form with pepsin, as shown in Figure 3(A), the HAECM hydrogel was a sol at room temperature, while crosslinked at 37 °C (Figure 3(B)). The crosslinked hydrogels were then frozen under certain conditions and lyophilized, achieving solid, stable, and highly porous scaffolds that preserve their size and morphology after rehydration. Scanning electron microscopy (SEM) of HAECM scaffolds (Figure 4(C,D)) revealed their interconnected porous structure and changes in porosity with alteration of ECM concentrations (5 mg/ml versus 8 mg/ml). The pore size ranged 100–300 μm, it showed that ECM concentration also affected pore size, a higher concentration of the ECM components resulted in smaller pores.

Figure 4(E,F) showed that the E_2_-MS dispersed in HAECM scaffolds uniformly, interconnecting with macroporous spongy structures. The structure of E_2_-MS would achieve the release of E_2_ by diffusing through these fiber network structures. The incorporation of E_2_-MS had no effect on the porous structure of scaffolds, which could also be confirmed by the porosity study (Table 2).

**Table 2.** Porosity of HAECM scaffolds with and without E_2_-MS (*n* = 4).

	Entrapment efficiency (%)
HAECM (8 mg/ml)	78.90 ± 5.61
HAECM (8 mg/ml)+30%E_2_-MS	75.12 ± 6.83
HAECM (5 mg/ml)	90.38 ± 4.87
HAECM (5 mg/ml)+30%E_2_-MS	88.15 ± 6.48

### 3.5. Characteristics of E_2_-loaded PLGA microspheres (E_2_-MS)

#### 3.5.1. Morphology and particle size

The surface morphology of E_2_-MS (502H) and E_2_-MS (503H) was examined visually by SEM. Photomicrographs of two PLGA microspheres loaded with E_2_ were shown in Figure 4(A,B). It was observed that the prepared drug-loaded microspheres were well sphere-shaped, homogeneous, and no crystalline of drugs or fragments of polymer exposed. The particles of PLGA microspheres were mainly 50–70 μm in diameter.

#### 3.5.2. Drug loading efficiency and encapsulation efficiency

As shown in Table 3, the loading percentage of E_2_ determined in E_2_-MS (502H) and E_2_-MS (503H) was estimated to be 8.18 ± 0.17% and 7.73 ± 0.14%, respectively. The encapsulation percentage of E_2_-MS (502H) and E_2_-MS (503H) was estimated to be 89.9 ± 6.61% and 85.1 ± 7.83%, respectively.

**Table 3.** Drug loading efficiency (LE) and encapsulation efficiency (EE) (*n* = 4).

Polymer	Drug loaded percent (%)	Entrapment efficiency (%)
PLGA (502H)	8.18 ± 0.17	89.90 ± 6.61
PLGA (503H)	7.73 ± 0.14	85.1 ± 7.83

### 3.6. In vitro drug release

**Figure 5.**
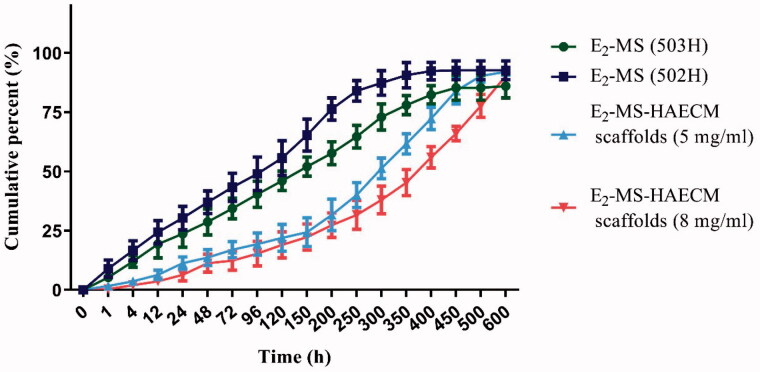
*In vitro* release profile of E_2_ from PLGA microspheres and microsphere-HAECM scaffolds as a function time in PBS (pH 7.4) at 37 ± 0.5 °C. Data are presented as mean ± standard deviation; *n* = 3.

*In vitro* drug release feature was important for characterizing the sustained released drug delivery system. As displayed in Figure 5, there was a certain burst drug release of E_2_-MS (502H) (about 30.33%) and E_2_-MS (503H) (23.67%) in 24 h. About 94.7% and 86.0% of E_2_ was released from E_2_-MS (502H) and E_2_-MS (503H) at the end of detection time. Figure 5 further confirmed the potential sustained release feature of the prepared scaffolds, the burst release rate was reduced, and the release rate in every stage was slower than E_2_-MS. Furthermore, the sustained-release lasted for about 21 days (94.3%) in E_2_-MS-HAECM scaffolds (5 mg/ml) group.

### 3.7. The proliferation of endometrial cell on HAECM scaffolds

**Figure 6.**
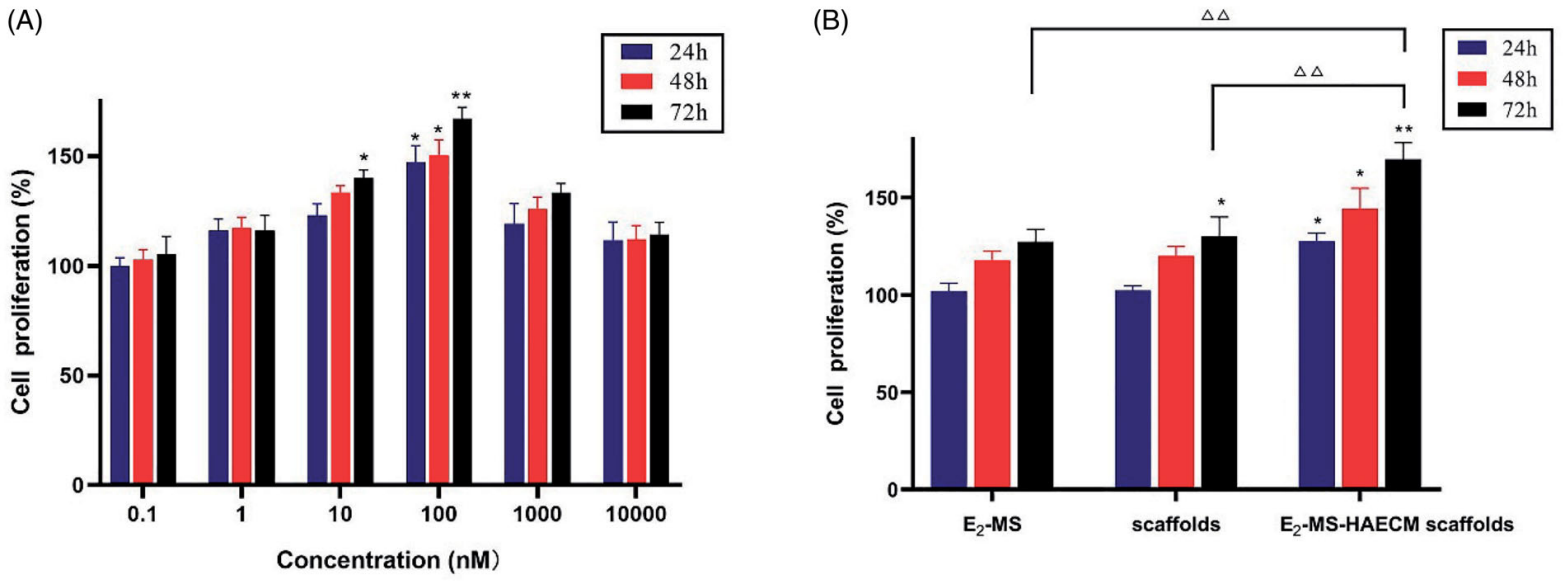
(A) Ishikawa cells were treated with different concentrations of E_2_ solution for 24 h, 48 h, and 72 h. Then, the cell proliferation was assessed by the MTT assay. *p < .05, **p < .01 vs untreated control group. (B) Ishikawa cells were treated with different preparations for 24 h, 48 h, and 72 h. *p < .05, **p < .01 vs untreated control group, △△*p* < .01 vs E_2_-MS-scaffolds group in 72 h. Data are presented as mean ± standard deviation; *n* = 3.

To further investigate the ability of the HAECM scaffolds on supporting endometrial cells, the MTT assay was conducted. After incubation with different concentrations of E_2_ solution, the cell proliferation rate increased significantly at the concentrations of 100 nM in all set incubation time (*p* < .05) (Figure 6(A)). According to the guidance of this result, 100 nM E_2_ was selected for subsequent experiments. As shown in Figure 6(B), compared to the control group, the significant proliferation of Ishikawa cells was noticed after treatment with E_2_-MS-HAECM scaffolds for 24, 48, and 72 h (*p* < .05). The proliferation rate in E_2_-MS-HAECM scaffolds group for 72 h was significantly higher than that in the other two groups (*p* < .05). When no E_2-_MS were added, the significant increase of proliferation rate in free HAECM scaffolds was observed compared to the control group in 72 h, indicating that the scaffolds offer a pleasant biocompatible environment for endometrial cell growth.

### 3.8. Effects of HAECM scaffolds on gene expression of cytokines and cytokine receptors

**Figure 7.**
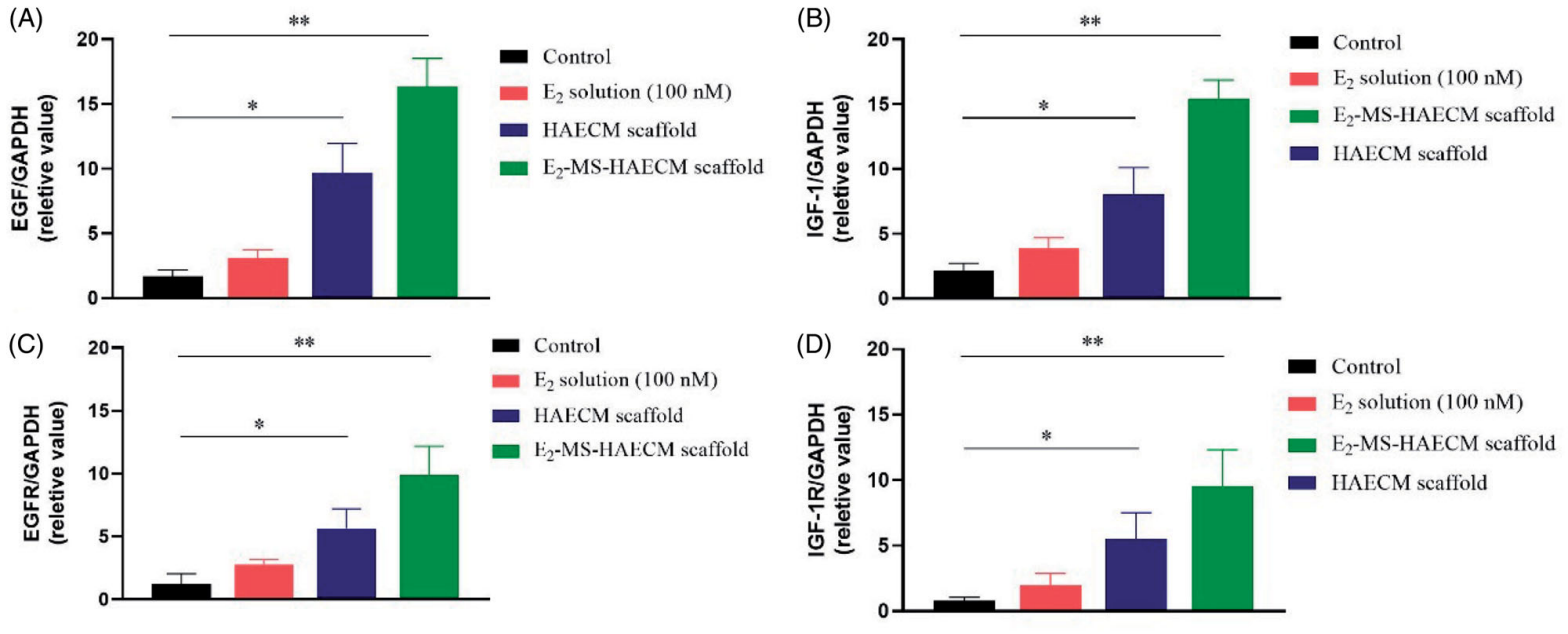
(A, B) Quantitative PCR analysis of EGF, IGF-1 mRNA levels in Ishikawa cells treated with different preparations after 72 h. *p < .05, **p < .01 vs control group. (C, D) Quantitative PCR analysis of EGFR, IGF-1R mRNA levels in Ishikawa cells treated with different preparations after 72 h. *p < .05, **p < .01 vs control group. Data are presented as mean ± standard deviation; *n* = 3.

After Ishikawa cells were incubated with different preparations for 72 h, the gene expressions of EGF, IGF-1, as well as their respective receptors (EGFR and IGF-1R) were analyzed. As shown in Figure 7(A,B), the mRNA levels of the cytokines EGF and IGF-1 were all increased in three exposed groups compared to the control group. The significant increase of both cytokines was observed in E_2_-MS-HAECM scaffolds group and HAECM scaffolds group (*p* < .05). The changes of EGFR and IGF-1R expression (Figure 7(C,D)) were consistent with the results of mRNA levels change in EGF and IGF-1.

## 4. Discussion

Bioactive scaffolds composed of extracellular matrix (ECM) are widely used in the field of medical science, such as tissue engineering and stem cell therapy. ECM scaffolds are prepared by decellularization of natural tissues and the ECM provides different soluble factors, including growth factors and cytokines that facilitate the restoration of regular structure and functional tissue (Dziki & Badylak, 2018; Nie & Wang, 2018).

Our study of decellularized amnion scaffolds integrated with the PLGA microsphere for endometrial cell growth is part of our laboratory’s research project and committed to developing biomaterial implants for application in IUAs. Because fertility issues are primary symptoms caused by severe IUAs, in consideration of female producibility and the safety of embryonic development, HAECM is considered as a potential multifunctional biomaterial for IUA treatment. When using the extracellular matrix for incubating cells or finally for implantation, it is essential to eliminate all antigens from tissues for preventing the immune response, while retaining the composition of the native ECM to the extent possible. How to achieve the balance between the preservation of essential bioactive substances and the elimination of all cellular components is the first problem we want to solve in this study. We demonstrated a new method of decellularization that AM can be completely decellularized without sodium dodecyl sulfate (SDS), which is one of the anionic surfactants, leading to adverse effects on cell viability and tissue structure (Figure 2(A)). The results indicated that the human amnion extracellular matrix (HAECM) is a promising cell-free biomaterial for physical support in the uterine cavity that also can be used as a scaffold for cell adhesion, migration, and growth. In our experiments, HAECM can be digested via pepsin to form a hydrogel with biophysical properties similar to collagen. HAECM hydrogel with different concentrations almost solidified within 32 min at 37 °C (Figure 3(C)). The components responsible for the gelation of HAECM are unknown but most likely gelation was due to the presence of self-assembling molecules such as collagens, laminins, and glycosaminoglycans (Aamodt & Grainger, 2016). Compared with synthetic copolymer hydrogels, which could be fabricated by chemical cross-linking agents, such as glutaraldehyde, NaOH, and ethylene glycol diglycidyl ether (Hennink & van Nostrum, 2012), the self-crosslinking behaviors of HAECM hydrogel could avoid the destruction of chemical crosslinking agents for proteins and peptides. In addition, the polymerization rate and mechanical strength could be further manipulated by altering the final ECM concentration and the source tissue from which the ECM hydrogel was prepared (Wolf et al., 2012).

Glycosaminoglycans (GAGs) are a class of long unbranched periodic negatively charged polysaccharides found in ECM that take part in many biological processes such as anticoagulation, angiogenesis and tissue regeneration (Walimbe & Panitch, 2019; Bojarski et al., 2020). They are partially responsible for the mechanical stability of tissues due to their capacity to retain a large quantity of water, enabling hydration of the ECM and rendering it resistant to compressive forces (Neves et al., 2020). Results of the GAGs and collagen assays indicated that the decellularization process did not lead to loss of collagen or GAG concentrations of the tissues. Furthermore, there was a statistically significant increase in these components of ECM compared to native AM; the main reason for this increase may be an elicitation of other soluble proteins and lipids constituents. Figure 4(C,D) exhibited morphology of the HAECM scaffolds with two different gel concentrations, both of them showed a porous network structure, which ensured the effective entrapment of PLGA microspheres and sequential release of drugs.

The scaffold had a pore size of 100–300 µm with good pore connectivity and a porosity of 79–90%, which was suitable for tissue penetration and excellent extended drug release. As ECM concentration could affect pore size and porosity (Table 2), distinct pore structures and sizes can be achieved by adjusting matrix concentration or using different freezing regimes (Soffer-Tsur et al., 2017). In subsequent studies, we are going to optimize the morphology of scaffolds to make it a better drug microrepository.

Next, O/W emulsion-solvent evaporation was used to prepare E_2_-MS. The SEM images (Figure 4(A,B)) of the lyophilized loading E_2_-MS powder presented a round sphere with a poreless surface. The uniform-sized particles not only enhanced drug delivery but also decreased the adverse effects of the delivered drugs. It was shown that the size of E_2_-MS made by 502H was smaller than that of E_2_-MS made by 503H. Due to the different molecular weight of polymers, the particle size is slightly different, which had no significant effect on drug loading and entrapment efficiency (Table 3). To retain the original microstructure of the scaffolds, PLGA microspheres (<100 μm) were taken. The composite of the scaffold with 30% E_2_-MS did not alter the shape and particle size of the microspheres, while the scaffold morphology slightly changed after the introduction of E_2_-MS, inferring that the addition of microspheres did not damage the porous structure of the scaffold (Figure 4(E,F)). With the addition of E_2_-MS, the decrease in porosity was not significant (from 78.9 ± 5.61% to 75.12 ± 6.83% by 8 mg/ml; from 90.38 ± 4.87% to 88.15 ± 6.48% by 5 mg/ml, respectively). For practical applications, the microsphere content should be controlled below 50% (Tan et al., 2009), so that the resulting scaffolds have large enough pores (>200 μm) and porosity (>90%). These characteristics are advantageous for cell infiltration, proliferation, and migration (Zhou et al., 2005).

To investigate the effects of a different combination of microspheres or microsphere-scaffold system in drug delivery, the drug release rate of E_2_-MS-HAECM complex scaffold were compared with E_2_-MS. Results showed the drug release rate of E_2_-MS was faster than the complex scaffolds (Figure 5). PLGA microsphere is a kind of excellent sustained-release drug delivery system for hydrophobic drugs. The release mechanisms include PLGA degradation and drug diffusion (Kamaly et al., 2016). In our study, we have used two PLGA materials with different molecular weight, 502H was a low-Mw polymer, while 503H was a higher-Mw polymer. Low-Mw polymer carrier release drugs are faster by faster degradation, which is consistent with the drug release result in Figure 5. According to the completeness of drug release from microspheres, E_2_-MS by 502H is more suitable. Based on this result, we chose E_2_-MS by 502H to composite with HAECM scaffolds in the follow-up study. It should be noted that the two PLGA microspheres have the phenomenon of burst release, which is 23.67% and 30.33% in the first 24 h release, respectively. The burst release was caused by the E_2_ located at the surface of MS. When MS was embedded in the scaffolds, E_2_ released at a slower rate than from free E_2_-MS. In the beginning, the burst release has been shown to be lowered due to the diffusional resistance caused by the scaffolds (Gu & Burgess, 2015), which maintain a neutral pH and are permeable to water and other small molecules. The scaffolds also provide a protective layer to maintain microsphere structure during drug release (Shen & Burgess, 2012). The E_2_ released from E_2_-MS-HAECM scaffold presented an initial burst release of 11.33 and 6.33% in the first 24 h, and then a relatively slower release up to 31.67 and 27.33% in the following 200 h. However, an increased E_2_ release occurred in the next 300 h, which can be attributed to the increased degradation of the HAECM scaffolds. Generally, a high degradation rate and swelling ratio of scaffold lead to a high diffusion rate of the MS from scaffolds, resulting in an increased drug release from the scaffold/MS (Sun et al., 2018). When the scaffold degraded completely, the drug release rate in the third stage mainly depends on the degradation of PLGA. In our study, the sustained-release of E_2_ lasted for about 21 days (94.3%) in E_2_-MS-HAECM scaffolds (5 mg/ml) group, which coincides with the secretion pattern of E_2_ during the female menstrual cycle. Compared with synthetic copolymer scaffolds (Rong et al., 2016; Zhang et al., 2018), the phenomenon of incomplete drug release has been improved. The increased concentration of scaffolds (8 mg/ml compared to 5 mg/ml) suggested reduced burst release and longer sustained release time due to their lower degradation rate of HAECM material *in vitro*. Therefore, the release profiles could be optimized by the properties of PLGA, the porosity, swelling, and degradation of the scaffolds based on the ECM concentrations and the ratio of MS/scaffolds. The controlled release of E_2_ from scaffolds was observed without a severe initial burst in 21 days, bringing the possibilities for improving the therapeutic effect for IUAs.

Furthermore, the ability of the HAECM scaffolds for supporting endometrial cells was evaluated. As shown in Figure 6(A), these cells require a medium supplemented with 100 nM E_2_; when given lower or higher E_2_ concentrations, the proliferation rate is lower, and the cell mortality rate is higher. The significant increase of proliferation rate in the free HAECM scaffolds showed excellent cytocompatibility of HAECM material, most cells exhibited normal and healthy morphology in the HAECM matrix. The results confirmed the manufactured scaffolds retain structural and functional properties required for cell growth *in vitro*. The highest proliferation rate was observed in E_2_-MS-HAECM scaffold in 72 h (Figure 6(B)), indicating the slow and stable release of E_2_ by the scaffolds is more conducive to the growth of endometrial cells than free E_2_. Therefore, the scaffolds could serve not only as a framework for physical supports but also as a comprehensive treatment device for regulating cell behaviors. Ding et al. developed a BMSCs loaded collagen scaffolds for the rat uterine regeneration after full-thickness injury (Ding et al., 2014). The BMSCs adhered to collagen scaffolds through interactions with the surface receptor, indicating a porous structure with suitable pore diameters, could promote the diffusion of oxygen and nutrients and the cell attachment.

To initially clarify the molecular mechanism of HAECM composite promoting endometrial growth, the following experiments focused on the gene expressions of EGF, IGF-1, and their respective receptors after exposure to treatment groups. Previous studies have reported that IGF-1 and EGF can promote the proliferation and migration of various types of cells, induce neovascularization to participate in tissue repair (Rijcken et al., 2014). As shown in Figure 7, when E_2_ solution was applied, the expression of EGF and IGF-1 did not change significantly, indicating that the effect of E_2_ on Ishikawa cells is independent of IGF-1 and EGF. However, the HAECM scaffold group and E_2_-MS-HAECM scaffold group increased mRNA levels of both cytokines significantly (*p* < .05). The results demonstrated EGF and IGF-1 are involved in endometrial proliferation, and HAECM partly promote the regeneration of endometrium through the EGF and IGF-1 family as an intermediary substance. Meanwhile, we found the expression of cytokines and their receptors peaked in E_2_-MS-HAECM scaffold group, suggesting the synergistic role of HAECM scaffold and E_2_ on endometrial regeneration, which was also supported by the results of cell proliferation (Figure 6).

Finally, the series of promising results *in vitro* need to be verified by animal experiments, additional experiments will be carried out to investigate therapeutic effects in IUA animal model, scaffold degradation rate *in vivo*, as well as the possible molecular mechanisms underlying the disease and healing.

## 5. Conclusions

Overall, this study describes an approach for the preparation of drug-delivering HAECM-derived scaffolds for endometrial cell growth. In the fabrication method, the addition of 30% E_2_-MS does not significantly modify the porous sponge structure of the scaffolds. Importantly, the composite improved the release properties of E_2_-MS by reducing the burst release. By adjusting the molecular weight of the PLGA polymer and the matrix concentration of the scaffolds, the goal of complete release in 21-day *in vitro* is achieved. Additionally, the cell proliferation experiments showed that slow and even release of E_2_ by the scaffolds is more conducive to the growth of endometrial cells than free E_2_. Therefore, more than creating a framework for simple mechanical supports, the advanced scaffolds provide higher functionality for the biologic guidance of endometrium regeneration.

## References

[CIT0001] Aamodt JM, Grainger DW. (2016). Extracellular matrix-based biomaterial scaffolds and the host response. Biomaterials 86:68–82. 26890039 10.1016/j.biomaterials.2016.02.003 PMC4785021

[CIT0002] Bojarski KK, Karczyńska AS, Samsonov SA. (2020). The role of glycosaminoglycans in procathepsin B maturation - molecular mechanism elucidated by a computational study. J Chem Inf Model 60:2247–56. 32155059 10.1021/acs.jcim.0c00023 PMC7588040

[CIT0003] Brown SB, Hankinson SE. (2015). Endogenous estrogens and the risk of breast, endometrial, and ovarian cancers. Steroids 99:8–10. 25555473 10.1016/j.steroids.2014.12.013

[CIT0004] Cai H, Qiao L, Song K, He Y. (2017). Oxidized, regenerated cellulose adhesion barrier plus intrauterine device prevents recurrence after adhesiolysis for moderate to severe intrauterine adhesions. J Minim Invasive Gynecol 24:80–8. 27742483 10.1016/j.jmig.2016.09.021

[CIT0005] Ding L, Li X, Sun H, et al. (2014). Transplantation of bone marrow mesenchymal stem cells on collagen scaffolds for the functional regeneration of injured rat uterus. Biomaterials 35:4888–900. 24680661 10.1016/j.biomaterials.2014.02.046

[CIT0006] Dreisler E, Kjer JJ. (2019). Asherman’s syndrome: current perspectives on diagnosis and management. Int J Womens Health 11:191–8. 30936754 10.2147/IJWH.S165474 PMC6430995

[CIT0007] Dziki JL, Badylak SF. (2018). Extracellular matrix for myocardial repair. Adv Exp Med Biol 1098:151–71. 30238370 10.1007/978-3-319-97421-7_8

[CIT0008] Gu B, Burgess DJ. (2015). Prediction of dexamethasone release from PLGA microspheres prepared with polymer blends using a design of experiment approach. Int J Pharm 495:393–403. 26325309 10.1016/j.ijpharm.2015.08.089 PMC4609624

[CIT0009] Hanstede MM, van der Meij E, Goedemans L, Emanuel MH. (2015). Results of centralized Asherman surgery, 2003-2013. Fertil Steril 104:1561-8.e1. 26428306 10.1016/j.fertnstert.2015.08.039

[CIT0010] Hennink WE, van Nostrum CF. (2012). Novel crosslinking methods to design hydrogels. Adv Drug Delivery Rev 64:223–36. 10.1016/s0169-409x(01)00240-x 11755704

[CIT0011] Kamaly N, Yameen B, Wu J, Farokhzad OC. (2016). Degradable controlled-release polymers and polymeric nanoparticles: mechanisms of controlling drug release. Chem Rev 116:2602–63. 26854975 10.1021/acs.chemrev.5b00346 PMC5509216

[CIT0012] Khan Z, Goldberg JM. (2018). Hysteroscopic management of Asherman’s syndrome. J Minim Invasive Gynecol 25:218–28. 29024798 10.1016/j.jmig.2017.09.020

[CIT0013] Kou L, Jiang X, Xiao S, et al. (2020). Therapeutic options and drug delivery strategies for the prevention of intrauterine adhesions. J Control Release 318:25–37. 31830539 10.1016/j.jconrel.2019.12.007

[CIT0014] Li C, Cai A, Sun C, et al. (2020). The study on the safety and efficacy of amnion graft for preventing the recurrence of moderate to severe intrauterine adhesions. Genes Dis 7:266–71. 32215296 10.1016/j.gendis.2019.03.003 PMC7083730

[CIT0015] McQuilling JP, Sanders M, Poland L, et al. (2019). Dehydrated amnion/chorion improves Achilles tendon repair in a diabetic animal model. Wounds 31:19–25. 30372415 PMC7989034

[CIT0016] Neves MI, Araujo M, Moroni L, et al. (2020). Glycosaminoglycan-inspired biomaterials for the development of bioactive hydrogel networks. Molecules 25:978. 10.3390/molecules25040978 PMC7070556 32098281

[CIT0017] Nie X, Wang DA. (2018). Decellularized orthopaedic tissue-engineered grafts: biomaterial scaffolds synthesised by therapeutic cells. Biomater Sci 6:2798–811. 30229775 10.1039/c8bm00772a

[CIT0018] Nogami M, Kimura T, Seki S, et al. (2016). A human amnion-derived extracellular matrix-coated cell-free scaffold for cartilage repair: in vitro and in vivo studies. Tissue Eng Part A 22:680–8. 27019057 10.1089/ten.TEA.2015.0285

[CIT0019] Portmann-Lanz CB, Ochsenbein-Kolble N, Marquardt K, et al. (2007). Manufacture of a cell-free amnion matrix scaffold that supports amnion cell outgrowth in vitro. Placenta 28:6–13. 16516964 10.1016/j.placenta.2006.01.004

[CIT0020] Rein DT, Schmidt T, Hess AP, et al. (2011). Hysteroscopic management of residual trophoblastic tissue is superior to ultrasound-guided curettage. J Minim Invasive Gynecol 18:774–8. 22024264 10.1016/j.jmig.2011.08.003

[CIT0021] Rijcken E, Sachs L, Fuchs T, et al. (2014). Growth factors and gastrointestinal anastomotic healing. J Surg Res 187:202–10. 24290527 10.1016/j.jss.2013.10.013

[CIT0022] Rong ZJ, Yang LJ, Cai BT, et al. (2016). Porous nano-hydroxyapatite/collagen scaffold containing drug-loaded ADM-PLGA microspheres for bone cancer treatment. J Mater Sci Mater Med 27:89. 26975746 10.1007/s10856-016-5699-0

[CIT0023] Shakouri-Motlagh A, Khanabdali R, Heath DE, Kalionis B. (2017). The application of decellularized human term fetal membranes in tissue engineering and regenerative medicine (TERM). Placenta 59:124–30. 28693892 10.1016/j.placenta.2017.07.002

[CIT0024] Shen J, Burgess DJ. (2012). Accelerated in vitro release testing of implantable PLGA microsphere/PVA hydrogel composite coatings. Int J Pharm 422:341–8. 22016033 10.1016/j.ijpharm.2011.10.020 PMC3246580

[CIT0025] Soffer-Tsur N, Peer D, Dvir T. (2017). ECM-based macroporous sponges release essential factors to support the growth of hematopoietic cells. J Control Release 257:84–90. 27671876 10.1016/j.jconrel.2016.09.021

[CIT0026] Sun X, Wang J, Wang Y, Zhang Q. (2018). Collagen-based porous scaffolds containing PLGA microspheres for controlled kartogenin release in cartilage tissue engineering. Artif Cells Nanomed Biotechnol 46:1957–66. 29103324 10.1080/21691401.2017.1397000

[CIT0027] Sun Y, Wang J, Zhang X, et al. (2008). Synchronic release of two hormonal contraceptives for about one month from the PLGA microspheres: in vitro and in vivo studies. J Control Release 129:192–9. 18539353 10.1016/j.jconrel.2008.04.022

[CIT0028] Tan H, Wu J, Lao L, Gao C. (2009). Gelatin/chitosan/hyaluronan scaffold integrated with PLGA microspheres for cartilage tissue engineering. Acta Biomater 5:328–37. 18723417 10.1016/j.actbio.2008.07.030

[CIT0029] Walimbe T, Panitch A. (2019). Proteoglycans in biomedicine: resurgence of an underexploited class of ECM molecules. Front Pharmacol 10:1661. 32082161 10.3389/fphar.2019.01661 PMC7000921

[CIT0030] Wolf MT, Daly KA, Brennan-Pierce EP, et al. (2012). A hydrogel derived from decellularized dermal extracellular matrix. Biomaterials 33:7028–38. 22789723 10.1016/j.biomaterials.2012.06.051 PMC3408574

[CIT0031] Zhang Q, Qin M, Zhou X, et al. (2018). Porous nanofibrous scaffold incorporated with S1P loaded mesoporous silica nanoparticles and BMP-2 encapsulated PLGA microspheres for enhancing angiogenesis and osteogenesis. J Mater Chem B 6:6731–43. 32254690 10.1039/c8tb02138d

[CIT0032] Zhang SS, Xia WT, Xu J, et al. (2017). Three-dimensional structure micelles of heparin-poloxamer improve the therapeutic effect of 17β-estradiol on endometrial regeneration for intrauterine adhesions in a rat model . Int J Nanomedicine 12:5643–57. 28848344 10.2147/IJN.S137237 PMC5557621

[CIT0033] Zhou Q, Gong Y, Gao C. (2005). Microstructure and mechanical properties of poly(L-lactide) scaffolds fabricated by gelatin particle leaching method. J Appl Polym Sci 98:1373–9.

